# Assessment of a three‐dimensional (3D) water scanning system for beam commissioning and measurements on a helical tomotherapy unit

**DOI:** 10.1120/jacmp.v16i1.4980

**Published:** 2015-01-08

**Authors:** Jean L. Peng, Michael S. Ashenafi, Daniel G. McDonald, Kenneth N. Vanek

**Affiliations:** ^1^ Department of Radiation Oncology Medical University of South Carolina Charleston SC USA

**Keywords:** beam data commissioning, 3D water scanning system, tomotherapy, small field dosimetry, quality assurance

## Abstract

Beam scanning data collected on the tomotherapy linear accelerator using the TomoScanner water scanning system is primarily used to verify the golden beam profiles included in all Helical TomoTherapy treatment planning systems (TOMO TPSs). The user is not allowed to modify the beam profiles/parameters for beam modeling within the TOMO TPSs. The authors report the first feasibility study using the Blue Phantom Helix (BPH) as an alternative to the TomoScanner (TS) system. This work establishes a benchmark dataset using BPH for target commissioning and quality assurance (QA), and quantifies systematic uncertainties between TS and BPH. Reproducibility of scanning with BPH was tested by three experienced physicists taking five sets of measurements over a six‐month period. BPH provides several enhancements over TS, including a 3D scanning arm, which is able to acquire necessary beam‐data with one tank setup, a universal chamber mount, and the OmniPro software, which allows online data collection and analysis. Discrepancies between BPH and TS were estimated by acquiring datasets with each tank. In addition, data measured with BPH and TS was compared to the golden TOMO TPS beam data. The total systematic uncertainty, defined as the combination of scanning system and beam modeling uncertainties, was determined through numerical analysis and tabulated. OmniPro was used for all analysis to eliminate uncertainty due to different data processing algorithms. The setup reproducibility of BPH remained within 0.5 mm/0.5%. Comparing BPH, TS, and Golden TPS for PDDs beyond maximum depth, the total systematic uncertainties were within 1.4 mm/2.1%. Between BPH and TPS golden data, maximum differences in the field width and penumbra of in‐plane profiles were within 0.8 and 1.1 mm, respectively. Furthermore, in cross‐plane profiles, the field width differences increased at depth greater than 10 cm up to 2.5 mm, and maximum penumbra uncertainties were 5.6 mm and 4.6 mm from TS scanning system and TPS modeling, respectively. Use of BPH reduced measurement time by 1–2 hrs per session. The BPH has been assessed as an efficient, reproducible, and accurate scanning system capable of providing a reliable benchmark beam data. With this data, a physicist can utilize the BPH in a clinical setting with an understanding of the scan discrepancy that may be encountered while validating the TPS or during routine machine QA. Without the flexibility of modifying the TPS and without a golden beam dataset from the vendor or a TPS model generated from data collected with the BPH, this represents the best solution for current clinical use of the BPH.

PACS number: 87.56.Fc

## I. INTRODUCTION

Helical TomoTherapy (TOMO) (Accuray, Sunnyvale, CA) delivers intensity‐modulated radiotherapy with a 6 MV linear accelerator mounted on a ring gantry. The unique geometry and radiation delivery characteristics of the TOMO machine create challenges for the physicist attempting to collect beam data.^1^, ^2^ For standard treatments, the accelerator revolves continuously while the treatment couch translates the patient through the 85 cm diameter gantry bore. The TOMO collimation system produces a flattening filter‐free (FFF) treatment fan beam 40 cm long in the lateral dimension (cross‐plane). The longitudinal field length (in‐plane) is selected by the user, and can be 5, 2.5, or 1 cm. Binary multileaf collimators move in the longitudinal direction, modulating the field throughout treatment.^(2)^ In addition to the distinct treatment geometry, beam modeling for the TOMO treatment planning system (TPS) is also unique. While a typical TPS generates a machine model based on data measured by the user, the TOMO TPS is preloaded with a machine model, based on a standard golden beam dataset.^3^, ^4^ This model cannot be adjusted by the users. However, it remains the users’ responsibility to verify that the factory “Standard” or “Golden” data accurately represents their specific machine. During acceptance and commissioning, and as part of a routine quality assurance (QA) program, the American Association of Physicists in Medicine (AAPM) Task Group 148^(5)^ (TG‐148) recommends measuring a static treatment beam to verify satisfactory agreement with the golden beam data in the TOMO TPS. Beam quality and profile agreement, both in‐ and cross‐ planes, are recommended for evaluation. In addition, because the beam quality changes continuously throughout the lifetime of the target and may exhibit significant dosimetric changes when the target is near end of life, PDD and profile agreement should also be verified annually as part of a comprehensive QA program. As noted above, the TOMO treatment geometry makes beam data collection with a full‐sized scanning system, designed for use with conventional linear accelerators, impossible.

During the initial product development, TomoTherapy (now Accuray Inc.) collaborated with Standard Imaging Inc. (Middleton, WI) to produce the TomoScanner (TS), a two‐dimensional (2D) scanning system customized for use within the TOMO geometry. The TS tank is reduced in size compared to the larger scanning tank normally used with conventional accelerators. This design allows the user to place the TS directly on the TOMO treatment couch and position it in the gantry bore for scanning. The TS 2D configuration allows for the measurement of depth‐dose information and beam profiles at multiple depths in a single direction. The TS is designed for use with Exradin (Standard Imaging, Middleton, WI) ionization chambers. The Exradin A1SL and A17 are provided by Accuray as the field and reference detectors, respectively. Standard Imaging provides software for controlling movement of the scanning arm, while Accuray provides Twin, a software program for data collection. Analysis of data collected with the TS must be done by exporting to third‐party software, such as Microsoft Excel. Prior to 2010, although two vendors (PTW Inc. and Standard Imaging Inc.) provided the scanning systems for TOMO, the TS was the only scanning system option from Accuray to new TOMO customers. The golden beam data used to generate the TOMO TPS beam model was originally measured using the TS. In addition, the machine specific factory benchmark measured data provided for each customer is also measured with TS system. Thus, the TS may seem to be the obvious choice for scanning the TOMO machine, but it also introduces the possibility of data bias.

In 2010, the Blue Phantom Helix (BPH) (IBA Dosimetry America, Bartlett, TN) scanning system was released commercially as an alternative to the TS. The BPH improves upon the TS in a number of ways. First, the BPH is a three‐dimensional (3D) scanner. This design allows for the collection of depth‐dose information and beam profiles at multiple depths in both the in‐ and cross‐plane directions with a single tank setup. The TS must be rotated to collect these profiles, resulting in added setup time and positional deviations. The added setup time is significant due to challenges created by the TOMO machine geometry. Because scanning tanks sit directly on the treatment couch and are slid on the couch into the gantry bore, table sag is a significant factor (approximately 10 mm or more), requiring additional adjustment of the tank position and leveling from inside the bore. Secondly, the BPH has a universal detector holder, allowing for greater measurement flexibility. Finally, the BPH utilizes the OmniPro Accept software program (IBA Dosimetry America, Inc.), for both scanning control and data analysis. These improvements make the BPH an attractive alternative to the TS. Currently, however, there are no publications examining reproducibility of the BPH system or the uncertainties a BPH user can expect when comparing data collected with the BPH to data collected with the TS or to the golden beam data in the TOMO TPS. In addition, Accuray has not provided benchmark‐measured beam data measured with the BPH scanning system for users.

The purpose of this work is to perform an experimental intercomparison of data collected with the BPH and TS systems, and the golden dataset present in the TOMO TPS. A benchmark BPH dataset is established, providing the user with the magnitude of uncertainty that can be reasonably expected when comparing data measured with the BPH to data measured with the TS or, ultimately, to golden data from the TOMO TPS. In addition, the reproducibility of setup and scanning with the BPH system is evaluated by analyzing five separate BPH datasets collected at separate times by three different physicists. With the data provided in this work, the clinical physicist will be able to use the BPH system to fulfill the recommendations outlined in AAPM TG‐148, with a full understanding of the capabilities of this system and the measurement discrepancy that should be expected compared to TS and TOMO TPS data.

## II. MATERIALS AND METHODS

### A. Water scanning system description

**Figure 1:**
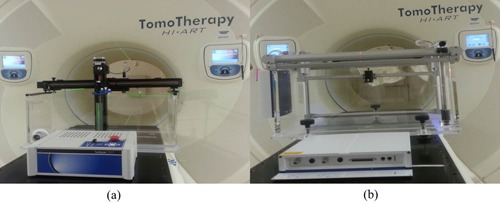
Schematic of the hardware setup for two water scanning systems in the tomotherapy (TOMO) machine: (a) Standard Imaging TomoScanner (TS) and (b) IBA Blue Phantom Helix (BPH).

The TS scanning tank has outer dimensions of approximately 32 cm (Height, H)×42 cm (Width, W)×69 cm (Length, L) and a scanning range of 28 cm (H)×60 cm (L). These dimensions allow for over scan of the 40 cm cross‐plane field at a depth of 20 cm. The 2D scanning arm is fitted with a lead screw motor providing 0.25 mm scanning resolution. The speed and distance of all motion are controlled by the TomoControl Unit (TCU). The field detector is an A1SL Exradin Miniature Shonka Thimble ionization chamber which has a collecting volume of 0.056 cm^3^ and an outside diameter of 6.25 mm. The reference detector is an A17 Exradin Slice Therapy ionization chamber which has a collecting volume of 1.91 cm^3^. The TomoElectrometer (TE) is an 8‐channel reference grade electrometer, with 1 fA resolution and accurately matched time constants. During data collection, two channels were connected with field and reference detectors. If needed, the TE can operate as a stand‐alone electrometer. The TS, TE, and TCU, shown in Fig. 1(a), are manufactured by Standard Imaging and provided by Accuray as the standard TOMO QA system.

The BPH is depicted in Fig. 1(b). The tank size (35 cm (H)×40.7 cm (W)×68 cm (L)) is designed to accommodate the limitations of TOMO treatment geometry discussed previously. A 3D scanning arm can collect beam data along all three axes and has a scanning range of 20 cm (H)×14 cm (W)×52 cm (L) and a positioning accuracy of ±0.1 mm. The scanning arm is fitted with a universal detector mount, allowing for the use of a detector of the user's choosing. For this study, a CC04 ionization chamber (IBA Dosimetry America, Bartlett, TN) with an active volume of 0.04 cm^3^ and a collecting volume diameter of 4 mm was selected. This detector was chosen due to its similarities to the Exradin A1SL. The BPH system is operated with the Common Control Unit (CCU) which integrates a controller and two independent electrometers in a compact design with precise scanning control. Additionally, the built‐in pressure and temperature sensors may be used for automatic corrections of absolute dosimetry measurements.

Characteristics of BPH and TS, including tank dimensions, scanning arm designs, positional accuracy, as well as detector and electrometer characteristics such as collection volume, active length, electrode material, supplied voltage, and charge/current range, are listed in Table 1.

**Table 1:** System characteristics of two water scanning system: Standard Imaging TomoScanner (TS) and IBA Blue Phantom Helix (BPH)

*Tank/System*	*Standard Imaging TomoScanner (TS)*	*IBA Blue Phantom Helix (BPH)*
arm motion	Two dimension (2D) Lateral (X) and Vertical (Z)	Three dimension (3D) Lateral (X), Longitudinal (Y), and Vertical (Z)
arm material	metal	aluminum
dimension (L×W×H) (cm)	69.1×41.9×32.3	68.0×40.7×35.0
scanning range (cm)	60.4 (L)×27.9 (H)	52.0 (L)×14.0 (W)×20.0 (H)
scanning arm leveling system	2 point arm screw	3 points micro
positioning accuracy	±0.25 mm	±0.1 mm
detector holder	specific (limited hold size/length)	universal
detector positioning	mechanical belt	magnetrostrictive senor
*Field Ion chamber*	*Exradin A1SL*	*Scanditronix CC04*
collecting volume (cm^3^)	0.057	0.04
outside diameter (mm)	6.4	4.8
inside diameter (mm)	4.0	4.0
cavity length (mm)	4.4	3.6
shell wall thickness (mm)	1.1	0.4
central electrode (material, size)	C552, 1 mm diameter	C552, 1 mm diameter
*Electrometer*	*TomoElectrometer*	*Common Control Unit (CCU)*
channel number 8	(1‐4 for general dosimetry)	2
supplied voltage	±150 V∼±304V	±50 V∼±500 V
charge range	0.01pC‐999,999nC	0.01pC‐999,999nC
current range	0.0001pA‐4.9nA	0.0005pA−4μA
sensitivity	10 msec	20 msec

### B. Water scanning software description

The TomoTherapy ElectroMeter Measurement System (TEMS) application is operated from the TomoTherapy dosimetry PC. It is used to control the operation of the TS scanning tank and collect profile data and provides tools to process and analyze profile data. The BPH system is controlled by the OmniPro Accept software program. The same program is used to perform data analysis. Both programs provide the all essential features, including tank and detector alignment, variable scanning speed/region, detector positioning both manually and remotely, bias support, background detection, automatic sequences for multiple scanning profiles and data analysis tools (e.g., renormalization, shifting, smoothing). The additional features of OmniPro which increase efficiency of data collection and analysis include:

1. Data analysis in real time. The multiple tab viewing allows the user to collect and analyze data at the same time, while the ability to overlap scans allows for qualitative comparisons. Physicists can discover differences from previous scans or commissioning data in real time so setup errors or machine problems can be detected immediately.
2. Numerical comparisons. Important machine parameters (such as field width, penumbra, maximum depth, and dose at specific depths) listed in AAPM TG reports can be defined and calculated immediately in OmniPro.
3. Flexible beam data format. Beam data measure in OmniPro can be converted into any format requested by TPSs or any third‐party software. In addition, the commissioning data from TS system or golden beam data from TOMO TPS can be converted into a format compatible with the OmniPro software.
4. Common software interface. The OmniPro software is the same software used for scanning traditional linear accelerators with the larger Blue Phantom 2 (IBA Dosimetry America, Bartlett, TN), thus eliminating the need for the physicists to master another, less frequently used, program.

### C. Acquired beam data collection and analysis

All beam scanning and data collection were performed in accordance with professional guidelines, including AAPM TG‐148^(5)^ and 106,^(6)^ which provide detailed recommendations on acceptance testing, beam commissioning, proper measurement techniques, and detector selection. The scanning tanks were leveled and positioned on the treatment couch with the TOMO isocenter placed at the surface of the water. Percent depth‐dose (PDD) measurements were performed for three jaw settings: $1.0\;\text{cm}\;({\text{Jaw}}_{1\text{cm}})$, $2.5\;\text{cm}\;({\text{Jaw}}_{2.5\text{cm}})$, and $5.0\;\text{cm}\;({\text{Jaw}}_{5\text{cm}})$. PDD measurement depths ranged from 0 to 20 cm. The lateral field width was fixed at 40 cm. The location of the point of measurement of each chamber was offset toward the radiation source by $0.6\;r_{\text{cav}}$ to account for the effective point of measurement. Beam profiles were acquired at depths of 1.5, 5, 10, 15, and 20 cm along in‐ and cross‐plane directions with each of the three jaw settings. User dependence and setup reproducibility of relative dose measurement with the BPH was tested by three experienced physicists taking five measurements over a six‐month period. Measurements from the BPH were compared against those from the TS.

All measurements were analyzed using the OmniPro software (version 7.3). Beam data was postprocessed before performing numerical comparisons. PDDs were smoothed by a least‐squares algorithm and renormalized to 100% at the depth of maximum dose ($d_{\text{max}}$). Profiles were smoothed by a median filter and then corrected for central axis discrepancies. Beam penumbra and field width were calculated for each profile. These parameters can be challenging to calculate for FFF beams. In‐plane TOMO profiles do not experience a large effect from the removal of the flattening filter due to their limited jaw settings. Therefore, traditional definitions of penumbra and field width are acceptable. Penumbra was defined as distance between 20% and 80% of the maximum field value, while field width was defined as the distance between points at 50% of the maximum filed value. Cross‐plane TOMO profiles, however, become significantly cone‐shaped in the absence of a flattening filter. For this reason, the standard definition of penumbra does not apply. Penumbra for cross‐plane profiles was quantitatively defined as the distance between 10% and 50% of the maximum profile value,^(1)^ which is considered as the rapid dose falloff region located at the TOMO FFF beam edge, while field width was defined as the distance between points at 25% of the maximum value. These points were chosen because they were the most reproducible for the TOMO FFF beam. For this study, renormalization of cross‐plane profiles was accomplished using the method proposed by Pönisch et al.^(7)^ This method renormalizes profiles to a ratio of the dose values at the inflection points in the penumbral regions between flattened and unflatten beams (approximately 50% of the central axis value for TOMO). After postprocessing, the beam data was tabulated and the two scanning systems were compared.

### D. Golden beam dataset in Helix Tomo Therapy treatment planning system (Golden TOMO TPS)

For the study, the golden beam data from the TPS was converted into the OmniPro format. This allowed for quantitative comparison of the golden TOMO TPS beam data with the data measured by BPH. Uncertainties caused by these differences were calculated and tabulated. As previously stated, the TPS golden beam dataset was originally measured with a TS system. Thus, some variations between BPH and golden beam data are expected, simply due to the use of different scanning systems. It should be noted, however, that discrepancy also exists between data measured by TS and TPS golden data.

The total systematic uncertainty, defined as the combination of scanning system and beam modeling uncertainties, was determined through numerical analysis and tabulated according to scan type. The discrepancies among the systems were quantified using mean and standard deviation (SD).

## III. RESULTS & DISCUSSION

### A. Reproducibility of beam data using BPH

**Table 2:** Reproducibility of measured beam data (PDDs) using BPH for ten repeated measurements from three experienced physicists over a period of six months

*Beam Data*	*Percent Depth Dose Curves Jaw Settings (cm)*	Mean±SD
$d_{\text{max}}$ (mm)	1	11.7±0.6
	2.5	11.7±0.5
	5	12.1±0.6
${\text{PDD}}_{5\text{cm}}(\%)$	1	78.8±0.3
	2.5	50.4±0.2
	5	82.5±0.2
${\text{PDD}}_{10\text{cm}}(\%)$	1	55.6±0.3
	2.5	57.4±0.2
	5	60.5±0.1
${\text{PDD}}_{20\text{cm}}(\%)$	1	28.1±0.1
	2.5	29.3±0.1
	5	31.8±0.1

Three experienced physicists used the BPH scanning system to measure five complete datasets of PDDs and profiles over a six‐month period. These measurements included scans using all three jaw settings (${\text{Jaw}}_{1\text{cm}}$, ${\text{Jaw}}_{2.5\text{cm}}$, and ${\text{Jaw}}_{5\text{cm}}$). The PDD curves were normalized to 100% at the corresponding depth of maximum dose for each jaw setting. All the measured PDDs, taken at different times and measured by different physicists, matched well. The average PDD values at 5, 10, and 20 cm depths, as well as the depths of maximum dose for each jaw setting, are summarized in Table 2 for easy comparison. As seen in the table, the maximum SD between the five sets of measurements was within 0.3 % for all three jaw settings. The overall SD of the $d_{\text{max}}$ for all jaw settings is 0.6 mm.

The mean field widths and penumbras for five sets of in‐plane and cross‐plane profiles are shown in Table 3. The maximum SD of the field width in the cross‐plane and in‐plane profiles was 0.4 mm and 0.1 mm, respectively. The mean penumbra in the cross‐plane was 0.7 mm, compared to 0.1 mm for the in‐plane profiles. The penumbra size was generally consistent for all three jaw settings. The fact that setup uncertainty is increased for cross‐plane profiles has been discussed in the literature.^5^, ^6^ This variance is due to the fact that the field width is much longer in the cross‐plane (40 cm) versus the in‐plane direction (maximum 5 cm). The increased uncertainty is demonstrated by greater variation in the measured field width and penumbras of the cross‐plane profiles compared to in‐plane profiles. In this study, user and setup variations using BPH scanning system had minimal effects on beam measurement precision.

**Table 3:** Reproducibility of measured beam data (profiles) using BPH for ten repeated measurements from three experienced physicists over a period of six months

*In‐plane Profiles*						*Cross‐plane Profiles*					
*Beam Data*	*Jaw Settings (cm)*	*Depth (cm)*	*Mean*	±	*SD*	*Beam Data*	*Jaw Settings (cm)*	*Depth (cm)*	*Mean*	±	*SD*
	1	1.5	11.2	±	0.1		1	1.5	411.0	±	0.2
		5	11.8	±	0.1			5	427.2	±	0.3
		10	12.5	±	0.1			10	450.7	±	0.3
		15	13.2	±	0.0			15	474.3	±	0.4
		20	13.9	±	0.0			20	498.2	±	0.4
Field Width (mm)	2.5	1.5	25.4	±	0.1	Field Width (mm)	2.5	1.5	411.3	±	0.3
		5	26.7	±	0.1			5	427.4	±	0.3
		10	28.4	±	0.1			10	450.8	±	0.3
		15	30.1	±	0.1			15	474.5	±	0.3
		20	31.7	±	0.1			20	498.3	±	0.3
	5	1.5	51.0	±	0.1		5	1.5	411.4	±	0.4
		5	53.2	±	0.0			5	426.5	±	0.3
		10	56.4	±	0.1			10	451.1	±	0.3
		15	59.7	±	0.0			15	474.8	±	0.1
		20	62.8	±	0.1			20	498.6	±	0.3
	1	1.5	4.4	±	0.0		1	1.5	19.4	±	0.4
		5	4.7	±	0.1			5	21.0	±	0.4
		10	5.1	±	0.0			10	25.7	±	0.5
		15	5.5	±	0.0			15	29.6	±	0.7
		20	5.9	±	0.1			20	34.7	±	0.9
Penumbra (mm)	2.5	1.5	5.3	±	0.0	Penumbra (mm)	2.5	1.5	16.4	±	0.6
		5	5.8	±	0.1			5	20.7	±	0.3
		10	6.5	±	0.1			10	25.4	±	0.7
		15	7.2	±	0.1			15	30.0	±	0.4
		20	7.7	±	0.1			20	34.3	±	0.5
	5	1.5	5.5	±	0.1		5	1.5	15.9	±	0.7
		5	6.3	±	0.1			21.2	±	0.3
		10	7.4	±	0.1			10	25.5	±	0.6
		15	8.4	±	0.1			15	30.8	±	0.5
		20	9.4	±	0.1			20	36.0	±	0.4

### B. Comparison to measured beam data using TS

Table 4 summarizes the differences seen between PDD parameters measured with BPH and TS systems. PDD curves were normalized to 100% at the depth of maximum dose ($d_{\text{max}}$) for the determination of PDD values at $5\;\text{cm}\;({\text{PDD}}_{d5\text{cm}})$, $10\;\text{cm}\;({\text{PDD}}_{d10\text{cm}})$, and $20\;\text{cm}\;({\text{PDD}}_{d20\text{cm}})$. Although PDD parameters are reasonably well‐matched between scanning systems, BPH shows a slightly increased $d_{\text{max}}$ and increased PDD values at depth compared to TS. For ${\text{Jaw}}_{1\text{cm}}$, ${\text{Jaw}}_{2.5\text{cm}}$, and ${\text{Jaw}}_{5\text{cm}}$, $d_{\text{max}}$, as measured by BPH, is increased by 1.3, 0.4, and 1.4 mm, respectively. The ${\text{PDD}}_{d10\text{cm}}$, ${\text{PDD}}_{d20\text{cm}}$, and the ratio of ${\text{PDD}}_{d20\text{cm}}$ to ${\text{PDD}}_{d10\text{cm}}$ varied by less than 1% for ${\text{Jaw}}_{2.5\text{cm}}$ and ${\text{Jaw}}_{5\text{cm}}$. However, ${\text{PDD}}_{d5\text{cm}}$ showed a maximum difference of 2.1% for ${\text{FW}}_{1\text{cm}}$. The likely cause of the minor PDD discrepancies is chamber volume effect^6^, ^8^ due to slight size differences between IBA CC04 used with the BPH and the Exradin A1SL used with TS system. The CC04 has an active volume of 0.04 cm^3^ compared to 0.053 cm^2^ for the A1SL. Chamber volume effects are most pronounced for small field sizes. For this reason, the largest differences between the BPH and TS system are seen in the PDD values for ${\text{Jaw}}_{1\text{cm}}$. Additionally, due to table sag caused by tank weight and TOMO special geometry, it is challenging for physicists to guarantee setup precision at the water surface inside the gantry bore of TOMO units. After detectors are set at the water surface located at the laser isocenter, the physicist has to re‐adjust the detector position at water surface after the heavy scanning tank is moved to radiation isocenter inside the gantry bore (700 mm in longitudinal direction away from laser isocenter). Therefore, tank setup uncertainty can contribute to the discrepancy seen between the two systems. PDDs at all three jaw settings show good qualitative agreement.

**Table 4:** Comparisons of the beam data variances between BPH and TS: PDDs

	*Percent Depth Dose Curves*			
*Beam Data*	*Jaw Settings (cm)*	*BPH*	*TS*	*BPH vs. TS*
d_max_(mm)	1	11.3	10.0	1.3
	2.5	11.4	11.0	0.4
	5	12.4	11.0	1.4
PDD_5cm_(%)	1	78.9	77.0	1.9
	2.5	80.5	79.3	1.2
	5	82.5	81.6	0.9
PDD_10cm_(%)	1	55.8	53.7	2.1
	2.5	57.5	56.7	0.8
	5	60.6	59.7	0.9
PDD_20cm_(%)	1	28.1	27.0	1.1
	2.5	29.3	28.7	0.6
	5	31.8	31.3	0.5
PDD_20cm_/PDD_10cm_	1	0.50	0.50	0.00
	2.5	0.51	0.51	0.00
	5	0.53	0.52	0.01

Table 5 shows a comparison of the measured field width and penumbras. The maximum differences in field width in the in‐ and cross‐plane profiles were 0.6 mm and 1.2 mm, respectively. The penumbra, as measured by BPH, was on average 0.6 mm and 2.8 mm larger in the in‐ and cross‐plane profiles compared to profiles measured with the TS system. As discussed previously, the normalization method proposed by Pönisch et al.^(7)^ was employed to allow for a direct qualitative comparison between profiles collected with BPH and TS.

The slight differences in field width and penumbra size can likely be explained by discrepancy in detector construction and orientation.^6^, ^8^ The BPH system measures in‐plane scans parallel to the axis of the chamber, while the TS requires the user to rotate the tank, resulting in in‐plane scans measured perpendicular to the axis of the chamber. This result in slightly sharper profiles from the TS system in the longitudinal direction and a reduced penumbra compared to BPH. In addition, the FFF characteristics of the cross‐plane profiles increase uncertainty in determining penumbra and field width, as discussed previously.

According to AAPM TG‐148,^(5)^ the standard beam data collected for beam commissioning includes PDDs and in‐ and cross‐plane profiles at 5 depths (1.5 cm, 5 cm, 10 cm, 15 cm, and 20 cm) for each jaw setting (${\text{Jaw}}_{1\text{cm}}$, ${\text{Jaw}}_{2.5\text{cm}}$, and ${\text{Jaw}}_{5\text{cm}}$). The average time needed to collect one standard dataset, including setup and scanning time, with BPH and TS were approximately 2 and 3 hrs, respectively. The 3D scanning arm of the BPH allows for the entire dataset to be collected with one tank setup. The TS, with its 2D scanning arm, requires the user to rotate and reposition the tank in order to collect in‐plane and cross‐plane profiles. This additional setup significantly increases the total time needed to collect a complete dataset. Additionally, the OmniPro software package available for the BPH allows for real‐time data analysis and comparison. The TS system requires the user to export data to third‐party software for analysis, increasing the probability of human error and adding to the time needed before the machine can be released for clinical use. Overall, the BPH significantly reduces the amount of time required and increases the collected data reliability for beam commissioning compared to TS.

**Table 5:** Comparisons of the beam data variances between BPH and TS: in‐ and cross‐profiles

*In‐plane Profiles*						*Cross‐plane Profiles*					
*Beam Data*	*Jaw Settings (cm)*	*Depth (cm)*	*BPH*	*TS*	*BPH vs. TS*	*Beam Data*	*Jaw Settings (cm)*	*Depth (cm)*	*BPH*	*TS*	*BPH vs. vs*.
	1	1.5	11.2	10.8	0.4		1	1.5	411.0	410.6	0.4
		5	11.9	11.3	0.6			5	427.2	426.4	0.8
		10	12.5	12.0	0.5			10	450.7	451.0	0.3
		15	13.2	12.8	0.4			15	474.3	474.8	0.5
		20	13.9	13.4	0.5			20	498.2	498.5	0.3
Field Width (mm)	2.5	1.5	25.4	25.4	0.0	Field Width (mm)	2.5	1.5	411.3	410.6	0.7
		5	26.6	26.6	0.0			5	427.4	427.5	0.1
		10	28.3	28.3	0.0			10	450.8	451.2	0.4
		15	30.1	29.9	0.2			15	474.5	475.0	0.5
		20	31.7	31.6	0.1			20	498.3	498.8	0.5
	5	1.5	51.0	50.9	0.1		5	1.5	411.4	411.0	0.4
		5	53.2	53.2	0.0			5	426.5	427.7	1.2
		10	56.4	56.6	0.2			10	451.1	451.6	0.5
		15	59.6	59.7	0.1			15	474.8	475.4	0.6
		20	62.8	62.9	0.1			20	498.6	499.1	0.5
	1	1.5	4.4	4.0	0.4		1	1.5	19.4	13.8	5.6
		5	4.7	4.3	0.4			5	21.0	17.1	3.9
		10	5.1	4.6	0.5			10	25.7	22.1	3.6
		15	5.5	5.0	0.5			15	29.6	26.7	2.9
		20	5.9	5.3	0.6			20	34.7	31.2	3.5
Penumbra (mm)	2.5	1.5	5.3	4.7	0.6	Penumbra (mm)	2.5	1.5	16.4	13.7	2.7
		5	5.8	5.2	0.6			5	20.7	17.7	3.0
		10	6.5	5.7	0.8			10	25.4	22.9	2.5
		15	7.2	6.3	0.9			15	30.0	27.8	2.2
		20	7.7	6.9	0.8			20	34.3	32.9	1.4
	5	1.5	5.5	5.0	0.5		5	1.5	15.9	13.9	2.0
		5	6.3	5.6	0.7			5	21.2	18.2	3.0
		10	7.3	6.6	0.7			10	25.5	23.9	1.6
		15	8.4	7.6	0.8			15	30.8	29.4	1.4
		20	9.4	8.7	0.7			20	36.0	33.5	2.5

### C. Comparison of TS measured beam data and TOMO TPS golden beam data

The qualitative comparison of PDD curves exported from the TPS versus those measured with TS for each available jaw size (${\text{Jaw}}_{1\text{cm}}$, ${\text{Jaw}}_{2.5\text{cm}}$, and ${\text{Jaw}}_{5\text{cm}}$ in TOMO unit) can be found in Table 6 including the $d_{\text{max}}$, PDD values at three different depths (5, 10, and 20 cm). TG‐148^(5)^ recommends performing quantitative analysis with ${\text{PDD}}_{d10\text{cm}}$ or the ration of ${\text{PDD}}_{d20\text{cm}}$ to ${\text{PDD}}_{d10\text{cm}}$. A comparison tolerance of 1% is recommended.^(5)^ Beyond $d_{\text{max}}$, the TOMO TPS data showed agreement within 1% compared to the TS scanning system for all FWs. The $d_{\text{max}}$ was within 1 mm for all jaw sizes. This excellent agreement is expected, since the TOMO TPS data was originally collected with a TS scanning system.

**Table 6:** Comparisons of the beam data variances from measured data using TS and golden beam data exported from TOMO TPS: PDDs

	*Percent Depth Dose Curves*			
$d_{\text{max}}$ (mm)	1	10.0	9.0	1.0
	2.5	11.0	11.0	0.0
	5	11.0	11.0	0.0
${\text{PDD}}_{5\text{cm}}(\%)$	1	77.0	77.4	0.4
	2.5	79.3	79.8	0.5
	5	81.6	82.2	0.6
${\text{PDD}}_{10\text{cm}}(\%)$	1	53.7	54.4	0.7
	2.5	56.7	57.0	0.3
	5	59.7	60.5	0.8
${\text{PDD}}_{20\text{cm}}(\%)$	1	27.0	27.4	0.4
	2.5	28.7	29.2	0.5
	5	31.3	32.1	0.8
${\text{PDD}}_{20\text{cm}}/{\text{PDD}}_{10\text{cm}}$	1	0.50	0.50	0.00
	2.5	0.51	0.51	0.00
	5	0.52	0.53	0.01

Constancy of the in‐plane profiles is of particular importance when delivering TOMO. Because the dose to the patient is the integration of the in‐plane profile shape with couch motion, the delivered dose could change by a significant amount with only a small change to profile shape or size. For example, when treating with the ${\text{Jaw}}_{1\text{cm}}$ with 1 cm collimator setting, the delivered dose could change by up to 10% if the in‐pane profile changes in width by only 1 mm.^(5)^ For this reason, TG‐148 recommends careful monitoring of the full width at half maximum (FWHM) for each jaw size, with a tolerance of 1% variation.^(5)^ Thus, the absolute tolerance is jaw setting dependent (0.5, 0.25, and 0.1 mm for the 5, 2.5, and 1 cm jaw settings, respectively). Table 7 lists penumbra and field width for selected in‐plane profiles. Penumbra and field width measurements agreed with TPS data within 0.5 mm. Due to the difficult nature of measuring field width and penumbra for FFF beams, TG‐148 recommends evaluating the consistency of the cross‐plane profiles by comparing measured profiles to golden beam data and calculating the average difference within the field core. The value corresponds to the average absolute difference for multiple off‐axis ratio measurements that are within the core of the beam. This average difference should be less than or equal to 1%,^(5)^ except at depths beyond 15 cm for ${\text{Jaw}}_{5\text{cm}}$, which has been verified in this study.

In addition, field width and penumbra of cross‐plane profiles were also evaluated, using the methods described previously and listed in Table 7. The average difference of field width between the measured data and the golden TPS data was within 1% of jaw size (40 cm). Field width and penumbra of cross‐plane profiles show greater variation than in‐plane profile, demonstrating the difficulty in accurately calculating these parameters for FFF beam profiles.

**Table 7:** Comparisons of the beam data variances from measured data using TS and golden beam data exported from TOMO TPS: in‐ and cross‐profiles

*In‐plane Profiles*						*Cross‐plane Profiles*					
*Beam Data*	*Jaw Settings (cm)*	*Depth (cm)*	*TS*	*TPS Golden*	*TS vs. TPS Golden*	*Beam Data*	*Jaw Settings (cm)*	*Depth (cm)*	*TS*	*TPS Golden*	*TS vs. TPS Golden*
	1	1.5	10.8	10.7	0.1		1	1.5	410.6	410.5	0.1
		5	11.3	11.3	0.0			5	426.4	427.1	0.7
		10	12.0	12.0	0.0			10	451.0	448.7	2.3
		15	12.8	12.7	0.1			15	474.8	472.7	2.1
		20	13.4	13.5	0.1			20	498.5	495.8	2.7
Field Width (mm)	2.5	1.5	25.4	25.4	0.0	Field Width (mm)	2.5	1.5	410.6	410.4	0.2
		5	26.6	26.6	0.0			5	427.5	427.1	0.4
		10	28.3	28.3	0.0			10	451.2	448.7	2.5
		15	29.9	30.1	0.2			15	475.0	472.7	2.3
		20	31.6	31.7	0.1			20	498.8	496.1	2.7
	5	1.5	50.9	51.0	0.1		5	1.5	411.0	410.9	0.1
		5	53.2	53.7	0.5			5	427.7	427.2	0.5
		10	56.6	56.9	0.3			10	451.6	448.7	2.9
		15	59.7	60.4	0.7			15	475.4	472.6	2.8
		20	62.9	63.3	0.4			20	499.1	496.1	3.0
	1	1.5	4.0	4.1	0.1		1	1.5	13.8	13.8	0.0
		5	4.3	4.4	0.1			5	17.1	16.6	0.5
		10	4.6	4.8	0.2			10	22.1	19.7	2.4
		15	5.0	5.1	0.1			15	26.7	22.9	3.8
		20	5.3	5.5	0.2			20	31.2	27.1	4.1
Penumbra (mm)	2.5	1.5	4.7	4.7	0.0	Penumbra (mm)	2.5	1.5	13.7	13.4	0.3
		5	5.2	5.1	0.1			5	17.7	17.3	0.4
		10	5.7	5.7	0.0			10	22.9	21.5	1.4
		15	6.3	6.2	0.1			15	27.8	25.7	2.1
		20	6.9	7.2	0.3			20	32.9	27.4	5.5
	5	1.5	5.0	5.1	0.1		5	1.5	13.9	12.8	1.1
		5	5.6	5.2	0.4			5	18.2	17.7	0.5
		10	6.6	6.2	0.4			10	23.9	23.7	0.2
		15	7.6	7.4	0.2			15	29.4	29.0	0.4
		20	8.7	9.6	0.9			20	33.5	28.9	4.6

### D. Total systematic uncertainties

Standard beam data measured with BPH were then directly compared to the TOMO TPS data as listed in Tables 8 and 9. In summary, BPH shows a slightly increased $d_{\text{max}}$ (up to 2.3 mm for ${\text{Jaw}}_{1\text{cm}}$) and increased PDD values (up to 1.5% for ${\text{Jaw}}_{1\text{cm}}$). The ratio of ${\text{PDD}}_{d20\text{cm}}$ to ${\text{PDD}}_{d10\text{cm}}$ varied by less than 0.5% for all jaw sizes. The maximum differences in field width in the in‐and cross‐plane profiles were 0.8 mm and 2.4 mm, respectively. The penumbra, as measured by BPH, was up to 1.1 mm and 7.6 mm larger in the in‐ and cross‐plane profiles, compared to profiles measured with the TOMO TPS golden data. Per vendor information and previous studies,^1^, ^3^ the TOMO TPS beam model is created through Monte Carlo deconvolution of data measured with the TS system. Because the underlying data was collected with the TS system, it is relatively straightforward to compare scans collected with the TS system in the clinic to the TPS model. Additionally, because remodeling is not a possibility in the TOMO TPS, and no golden dataset has been provided by the vendor for the BPH scanning system, comparing the TPS model to scans collected with the BPH system is slightly more challenging. Therefore, it is necessary to quantify the total uncertainty a BPH user can expect when using the BPH system to validate the TOMO TPS. The total uncertainty is a function of two separate uncertainty components. The first uncertainty component is due to tank and ion chamber discrepancy between BPH and the TS system. These discrepancies include tank material, positional accuracy, and ion chamber active volume and construction material. The second uncertainty component is caused by the Monte Carlo deconvolution method used to remove detector perturbation effects from the TPS model. The combination of these two components of uncertainty can be used to calculate the total uncertainty a user can expect when comparing data collected with the BPH to data from the TOMO TPS, as listed in Tables 10 and 11.

**Table 8:** Comparisons of the beam data variances from measured data using BPH and golden beam data exported from TOMO TPS: PDDs

	*Percent Depth Dose Curves*			
*Beam Data*	*Jaw Settings (cm)*	*BPH*	*TPS Golden*	*BPH vs. TPS Golden*
$d_{\text{max}}$ (mm)	1	11.3	9.0	2.3
	2.5	11.4	11.0	0.4
	5	12.4	11.0	1.4
${\text{PDD}}_{5\text{cm}}(\%)$	1	78.9	77.4	1.5
	2.5	80.5	79.8	0.7
	5	82.5	82.2	0.3
${\text{PDD}}_{10\text{cm}}(\%)$	1	55.8	54.4	1.4
	2.5	57.5	57.0	0.5
	5	60.6	60.5	0.1
${\text{PDD}}_{20\text{cm}}(\%)$	1	28.1	27.4	0.7
	2.5	29.3	29.2	0.1
	5	31.8	32.1	0.3
${\text{PDD}}_{20\text{cm}}/{\text{PDD}}_{10\text{cm}}$	1	0.50	0.50	0.00
	2.5	0.51	0.51	0.00
	5	0.53	0.53	0.00

**Table 9:** Comparisons of the beam data variances from measured data using BPH and golden beam data exported from TOMO TPS: in‐ and cross‐profiles

*In‐plane Profiles*						*Cross‐plane Profiles*					
*Beam Data*	*Jaw Settings (cm)*	*Depth (cm)*	*BPH*	*TPS Golden*	*BPH vs. TPS Golden*	*Beam Data*	*Jaw Settings (cm)*	*Depth (cm)*	*BPH*	*TPS Golden*	*BPH vs. TPS Golden*
	1	1.5	11.2	10.7	0.5		1	1.5	411.0	410.5	0.5
		5	11.9	11.3	0.6			5	427.2	427.1	0.1
		10	12.5	12.0	0.5			10	450.7	448.7	2.0
		15	13.2	12.7	0.5			15	474.3	472.7	1.6
		20	13.9	13.5	0.4			20	498.2	495.8	2.4
Field Width (mm)	2.5	1.5	25.4	25.4	0.0	Field Width (mm)	2.5	1.5	411.3	410.4	0.9
		5	26.6	26.6	0.0			5	427.4	427.1	0.3
		10	28.3	28.3	0.0			10	450.8	448.7	2.1
		15	30.1	30.1	0.0			15	474.5	472.7	1.8
		20	31.7	31.7	0.0			20	498.3	496.1	2.2
	5	1.5	51.0	51.0	0.0		5	1.5	411.4	410.9	0.5
		5	53.2	53.7	0.5			5	426.5	427.2	0.7
		10	56.4	56.9	0.5			10	451.1	448.7	2.4
		15	59.6	60.4	0.8			15	474.8	472.6	2.2
		20	62.8	63.3	0.5			20	498.6	496.1	2.5
	1	1.5	4.4	4.1	0.3		1	1.5	19.4	13.8	5.6
		5	4.7	4.4	0.3			5	21.0	16.6	4.4
		10	5.1	4.8	0.3			10	25.7	19.7	6.0
		15	5.5	5.1	0.4			15	29.6	22.9	6.7
		20	5.9	5.5	0.4			20	34.7	27.1	7.6
Penumbra (mm)	2.5	1.5	5.3	4.7	0.6	Penumbra (mm)	2.5	1.5	16.4	13.4	3.0
		5	5.8	5.1	0.7			5	20.7	17.3	3.4
		10	6.5	5.7	0.8			10	25.4	21.5	3.9
		15	7.2	6.2	1.0			15	30.0	25.7	4.3
		20	7.7	7.2	0.5			20	34.3	27.4	6.9
	5	1.5	5.5	5.1	0.4		5	1.5	15.9	12.8	3.1
		5	6.3	5.2	1.1			5	21.2	17.7	3.5
		10	7.3	6.2	1.1			10	25.5	23.7	1.8
		15	8.4	7.4	1.0			15	30.8	29.0	1.8
		20	9.4	9.6	0.2			20	36.0	28.9	7.1

**Figure 2:**
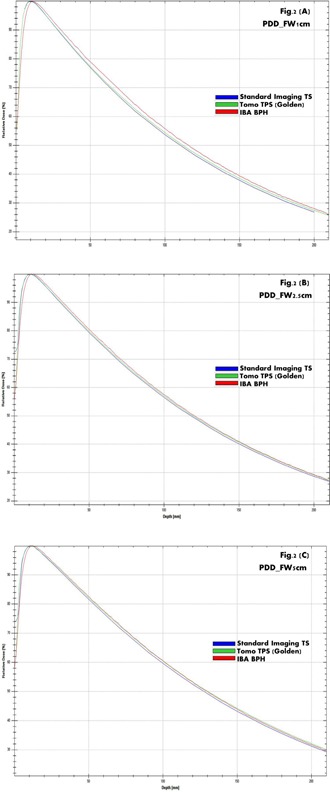
Percent depth dose (PDD) overlap for each jaw settings among TS, tomotherapy treatment planning golden (TOMO TPS Golden), and BPH: (a) ${\text{Jaw}}_{1\text{cm}}$, (b) ${\text{Jaw}}_{2.5\text{cm}}$, and (c) ${\text{Jaw}}_{5\text{cm}}$.

PDD curves consistently show in Fig. 2 among BPH, TS, and TOMO golden TPS a higher uncertainty due to tank discrepancy compared to differences caused by TPS deconvolution. This is expected, because chamber perturbation and volume effects are relatively limited for depth dose scans. Uncertainty due to differences between the BPH and TS systems is larger, driven by differences in tank material and size, as well as scanning arm and chamber differences. 3, 4, 5 provide a qualitative comparison of profiles among BPH, TS, and TOMO golden TPS. For in‐plane profiles, uncertainty due to tank differences was again larger than TPS uncertainty. The primary cause of tank discrepancy in longitudinal direction is likely the scan direction of the TS compared to BPH. The TS system forces the user to rotate the tank, resulting in scans taken perpendicular to the long axis of the chamber. The BPH allows for a single tank setup; however, longitudinal scans are taken parallel to the long axis of the chamber, which can result in slightly increased penumbra. No profile showed an uncertainty of either type larger than 1 mm. Cross‐plane profiles showed a greater amount of TPS uncertainty compared to scanning system uncertainty. TPS data had a reduced field width and significantly reduced penumbra compared to the TS measured data, likely due to chamber volume effects, which would be larger for profiles of this shape. Because cross‐plane scans are measured perpendicular to the chamber axis for both the TS and BPH, discrepancy due to the scanning system is reduced. Specifically, field size uncertainties due to differences between the scanning systems are within 1 mm, while TPS uncertainty is approximately 3 mm, on average. Penumbra differences from scanning system uncertainty are less than 1 mm and up to 10 mm from TPS uncertainty.

**Table 10:** Total systematic uncertainties from scanning system and TPS modeling: PDDs

*Percent Depth Dose Curves*				
*Beam Data*	*Jaw Settings (cm)*	*Scanning System Uncertainties (A)*	*TPS Modeling Uncertainties (B)*	Systematic Uncertainties=(A)±(B)
$d_{\text{max}}$ (mm)	1	1.3	1.0	1.3±1.0
	2.5	0.4	0.0	0.4±0.0
	5	1.4	0.0	1.4±0.0
${\text{PDD}}_{5\text{cm}}(\%)$	1	1.9	0.4	1.9±0.4
	2.5	1.2	0.5	1.2±0.5
	5	0.9	0.6	0.9±0.6
${\text{PDD}}_{10\text{cm}}(\%)$	1	2.1	0.7	2.1±0.7
	2.5	0.8	0.3	0.8±0.3
	5	0.9	0.8	0.9±0.8
${\text{PDD}}_{20\text{cm}}(\%)$	1	1.1	0.4	1.1±0.4
	2.5	0.6	0.5	0.6±0.5
	5	0.5	0.8	0.5±0.8
${\text{PDD}}_{20\text{cm}}/{\text{PDD}}_{10\text{cm}}$	1	0.0	0.0	0.0±0.0
	2.5	0.0	0.0	0.0±0.0
	5	0.0	0.0	0.0±0.0

**Table 11:** Total systematic uncertainties from scanning system and TPS modeling: profiles

*In‐plane Profiles*					
*Beam Data*	*Jaw Settings (cm)*	*Depth (cm)*	*Scanning System Uncertainties (A)*	*TPS Modeling Uncertainties (B)*	Systematic Uncertainties=(A)±(B)
	1	1.5	0.4	0.1	0.4±0.1
		5	0.6	0.0	0.6±0.0
		10	0.5	0.0	0.5±0.0
		15	0.4	0.1	0.4±0.1
		20	0.5	0.1	0.5±0.1
Field Width (mm)	2.5	1.5	0.0	0.0	0.0±0.0
		5	0.0	0.0	0.0±0.0
		10	0.0	0.0	0.0±0.0
		15	0.2	0.2	0.2±0.2
		20	0.1	0.1	0.1±0.1
	5	1.5	0.1	0.1	0.1±0.1
		5	0.0	0.5	0.0±0.5
		10	0.2	0.3	0.2±0.3
		15	0.1	0.7	0.1±0.7
		20	0.1	0.4	0.1±0.4
	1	1.5	0.4	0.1	0.4±0.1
		5	0.4	0.1	0.4±0.1
		10	0.5	0.2	0.5±0.2
		15	0.5	0.1	0.5±0.1
		20	0.6	0.2	0.6±0.2
Penumbra (mm)	Penumbra (mm)	1.5	0.6	0.0	0.6±0.0
		5	0.6	0.1	0.6±0.1
		10	0.8	0.0	0.8±0.0
		15	0.9	0.1	0.9±0.1
		20	0.8	0.3	0.8±0.3
	5	1.5	0.5	0.1	0.5±0.1
		5	0.7	0.4	0.7±0.4
		10	0.7	0.4	0.7±0.4
		15	0.8	0.2	0.8±0.2
		20	0.7	0.9	0.7±0.9
*Cross‐plane Profiles*					
	1	1.5	0.4	0.1	0.4±0.1
		5	0.8	0.7	0.8±0.7
		10	0.3	2.3	0.3±2.3
		15	0.5	2.1	0.5±2.1
		20	0.3	2.7	0.3±2.7
Field Width (mm)	2.5	1.5	0.7	0.2	0.7±0.2
		5	0.1	0.4	0.1±0.4
		10	0.4	2.5	0.4±2.5
		15	0.5	2.3	0.5±2.3
		20	0.5	2.7	0.5±2.7
	5	1.5	0.4	0.1	0.4±0.1
		5	1.2	0.5	1.2±0.5
		10	0.5	2.9	0.5±2.9
		15	0.6	2.8	0.6±2.8
		20	0.5	3.0	0.5±3.0
	1	1.5	5.6	0.0	5.6±0.0
		5	3.9	0.5	3.9±0.5
		10	3.6	2.4	3.6±2.4
		15	2.9	3.8	2.9±3.8
		20	3.5	4.1	3.5±4.1
Penumbra (mm)	2.5	1.5	2.7	0.3	2.7±0.3
		5	3.0	0.4	3.0±0.4
		10	2.5	1.4	2.5±1.4
		15	2.2	2.1	2.2±2.1
		20	1.4	5.5	1.4±5.5
	5	1.5	2.0	1.1	2.0±1.1
		5	3.0	0.5	3.0±0.5
		10	1.6	0.2	1.6±0.2
		15	1.4	0.4	1.4±0.4
		20	2.5	4.6	2.5±4.6

**Figure 3:**
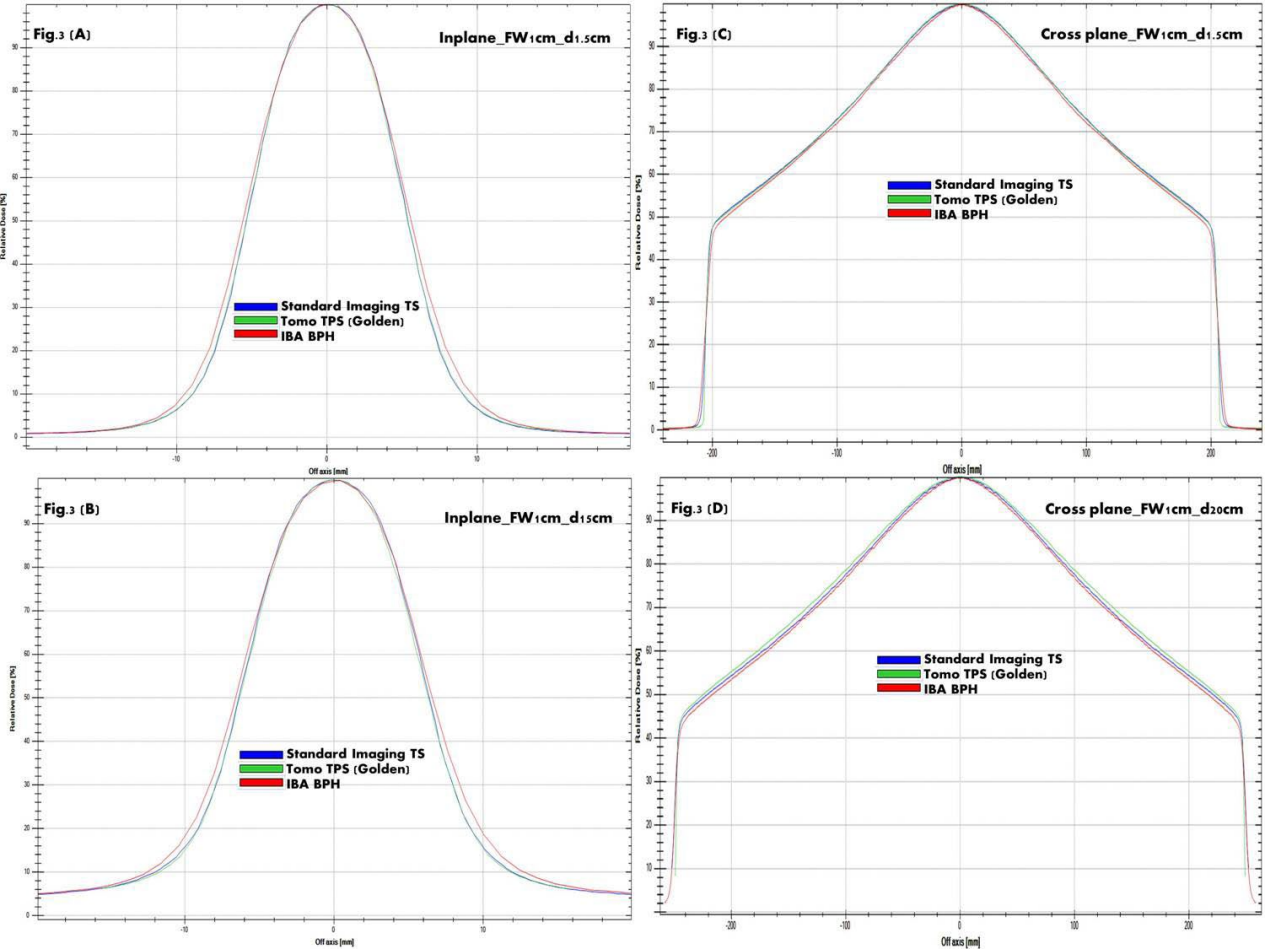
The examples of TS, Tomo TPS Golden, and BPH profiles overlap at two depths are shown for ${\text{Jaw}}_{1\text{cm}}$. In‐plane profiles at depth $1.5\;\text{cm}\;(d_{1.5\text{cm}})$ and $15\;\text{cm}\;(d_{15\text{cm}})$ are shown in (a) and (b), respectively. Cross‐plane profiles at $d_{1.5\text{cm}}$ and $d_{20\text{cm}}$ are shown in (c) and (d), respectively.

**Figure 4:**
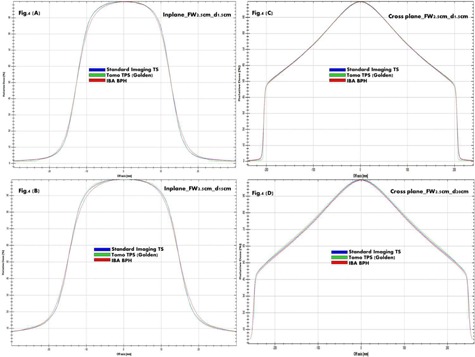
The examples of TS, Tomo TPS Golden, and BPH profiles overlap at two depths are shown for ${\text{Jaw}}_{2.5\text{cm}}$. In‐plane profiles at depth $1.5\;\text{cm}\;(d_{1.5\text{cm}})$ and $15\;\text{cm}\;(d_{15\text{cm}})$ are shown in (a) and (b), respectively. Cross‐plane profiles at $d_{1.5\text{cm}}$ and $d_{20\text{cm}}$ are shown in (c) and (d), respectively.

**Figure 5:**
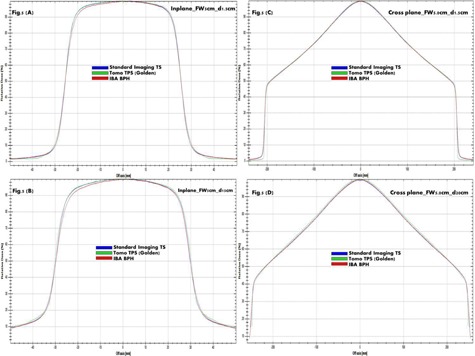
The examples of TS, Tomo TPS Golden, and BPH profiles overlap at two depths are shown for ${\text{Jaw}}_{5\text{cm}}$. In‐plane profiles at depth $1.5\;\text{cm}\;(d_{1.5\text{cm}})$ and $15\;\text{cm}\;(d_{15\text{cm}})$ are shown in (a) and (b), respectively. Cross‐plane profiles at $d_{1.5\text{cm}}$ and $d_{20\text{cm}}$ are shown in (c) and (d), respectively.

## IV. CONCLUSIONS

The BPH scanning system is an efficient and reliable way to collect beam data on a TOMO unit. The 3D scanning arm and OmniPro software with online analysis allow for significant savings in scanning time, compared to the TS system. The user is not able to change the TOMO TPS model, generated with data collected using TS; therefore, it is necessary to determine what uncertainties a user should expect when validating the TOMO TPS with the BPH. This work has provided the complete benchmark data using BPH and quantifies the amount of uncertainties between BPH, TS, and TOMO TPS. With this data, a physicist can utilize the BPH in a clinical setting with an understanding of the scan discrepancy he or she may encounter while validating the TPS or during routine machine QA. Without the flexibility of modifying the TPS and without a golden beam dataset from the vendor or TPS model generated from data collected with the BPH, this represents the best solution for current clinical use of the BPH.

## Supporting information

Supplementary Material
