# Supplementary material for: Assessment of a three‐dimensional (3D) water scanning system for beam commissioning and measurements on a helical tomotherapy unit
Source: J Appl Clin Med Phys. 2015 Jan 8;16(1):51–68. doi: 10.1120/jacmp.v16i1.4980 (PMC5689986; doi:10.1120/jacmp.v16i1.4980)
Supplement: Supplementary file 1 — Supplementary Material [file ACM2-16-051-s001.docx]

**Assessment of a three dimensional (3-D) water scanning system for beam commissioning and measurements on a helical tomotherapy unit**

Jean L. Peng^1^, Michael S. Ashenafi^1^, Daniel G. McDonald^1^, Kenneth N. Vanek^1^

^1^Department of Radiation Oncology, Medical University of South Carolina

*169 Ashley Ave., Charleston, South Carolina, 29425*

[*pengl@musc.edu*](mailto:pengl@musc.edu)*,,* *ashenafi@musc.edu,* *mcdonad@musc.edu* , *vanek@musc.edu*

Address correspondence to:

Jean L. Peng, Ph.D.

Department of Radiation Oncology, Medical University of South Carolina

*169 Ashley Ave, Charleston, South Carolina, 26425*

*E-mail:* [*pengl@musc.edu*](mailto:pengl@musc.edu)

Running Title: Assessment of the novel water scanning system on tomotherapy
